# Important Role of Intestinal Microbiota in Chemotherapy-induced Diarrhea and Therapeutics

**DOI:** 10.7150/jca.99421

**Published:** 2025-01-01

**Authors:** Wanrou Jiang, Yongjun Wu, Xiuyun He, Ling Jiang, Wanyi Zhang, Wenjuan Zheng, Min Hu, Chaofu Zhu

**Affiliations:** Oncology Department I, Hospital of Chengdu University of Traditional Chinese Medicine.

**Keywords:** chemotherapy, chemotherapy-induced diarrhea, intestinal microbiota, intestinal mucositis, intestinal barrier

## Abstract

Chemotherapy-induced diarrhea (CID) is a common and harmful side effect of chemotherapy, greatly impacting patients' quality of life and potentially compromising their chances of survival. Disruption of the balance in intestinal microbiota and compromised integrity of the intestinal barrier are key factors contributing to CID caused by mucositis. This paper investigated the mechanism through which intestinal microbiota activate Toll-like receptors and STING pathways to mediate intestinal mucosal inflammation resulting from immune responses in the gut, uncovering a novel mechanism of intestinal microbiota in chemotherapy-induced diarrhea, and suggesting innovative approaches for the prevention and management of CID.

## Introduction

Chemotherapy is a crucial treatment for malignant tumors, but chemotherapy-induced diarrhea (CID) is a common and severe side effect. CID can occur during or after chemotherapy, with symptoms ranging from mild abdominal pain and increased bowel movements to severe cases lasting several days 1. The NCI CTCAE 5.0 categorizes CID into grades 1 to 5, with grade 1 being mild and grade 5 being fatal 2. Studies show that 50% to 80% of patients on irinotecan and fluorouracil experience CID, with a significant portion suffering severe cases (grades 3 or 4) 3. This severe form often disrupts cancer treatment, leading to dose reductions, delays, or cessation in about 60% of patients 4. CID also contributes to approximately 1% mortality 5.

Current insights indicate that chemotherapy and metabolic byproducts influence various elements of the intestinal barrier, such as the intestinal epithelium, goblet cells, mucus layer, and immune system. This interaction triggers an immune response that leads to inflammation of the intestinal mucosa, heightened permeability to harmful substances, increased secretion within the intestines, diminished water absorption in both stomach and intestines, accumulation of undigested materials in the lumen, and a resulting permeability gradient. Collectively, these processes result in water influx into the intestinal lumen, ultimately causing diarrhea 6-7. Current treatments like loperamide, atropine, and octreotide manage symptoms by suppressing motility but can cause adverse effects and do not address microbiota or barrier function. Thus, novel or alternative therapies that minimize side effects and improve treatment safety are needed. This paper will explore how intestinal microbiota influences CID development and its clinical applications.

## Chemotherapy-induced diarrhea and intestinal microbiota

### Intestinal microbiota

The human intestinal microbiota is a bacterial organ composed of more than 10^14^ microorganisms. It plays a crucial role in maintaining human health and promoting microecological balance within the body by modulating the permeability of intestinal epithelial cells and eliciting metabolic and immune responses 8-10. The normal intestinal microbiota maintains human health and microecological balance in the body. Numerous studies have demonstrated the pivotal role of the intestinal microbiota in the pathogenesis of intestinal mucositis 11-14.

The gut microbiota, residing within the human body, actively engages in diverse physiological and pathological processes by metabolizing and synthesizing carbohydrates, organic acids, as well as specific microbial metabolites such as vitamins and short-chain fatty acids (SCFAs). These activities encompass nutrient absorption, immune homeostasis regulation, as well as the etiology and progression of diseases 15.

### Chemotherapy-induced intestinal microbiota dysbiosis

The human intestinal microbiota is mostly composed of Proteobacteria, Firmicutes, Actinobacteria, and Bacteroidetes phyla 16, 17. Phylum Firmicutes and Bacteroidetes collectively account for 90% of the total intestinal microbiota 18. Within phylum Firmicutes, families Bacillaceae and Clostridiaceae are the predominant group, while family Prevotellaceae constitute the major genera within phylum Bacteroidetes. Phylum Actinobacteria are mainly represented by family Mycobacteriaceae.

Multiple clinical trials and animal studies have demonstrated that chemotherapy drugs can disrupt the homeostasis of the gut microbiome through hepatic metabolism or enterohepatic circulation. Montassier analyzed fecal samples from 28 non-Hodgkin lymphoma patients after chemotherapy and found a decrease in the relative abundance and disruption in the structure of the gut microbiota. Firmicutes and Actinobacteria showed decreased relative abundance, while Proteobacteria exhibited a significant increase 19. Sophie Viaud observed an increased relative abundance of gram-negative bacteria in the gut microbiota of rats treated with 5-fluorouracil (5-FU), along with an increased migration of intestinal microbiota to cervical and mesenteric lymph nodes 20. Margot Fijlstra's research revealed that in a rat model of gastrointestinal mucositis induced by methotrexate (MTX), there was an increase in intestinal inflammation levels, a decrease in villus length, and a reduction in the relative abundance of most bacterial genera within the gut microbiota. Additionally, there was a decrease in the relative abundance of streptococci but an increase in the relative abundance of Bacteroides 21.

Despite variations in chemotherapy regimens, there are certain similarities in their impact on the gut microbiota. These include an increase in Firmicutes abundance, a decrease in Bacteroidetes' abundance, an increase in gram-negative (G-) bacteria such as *E. coli* and Pseudomonas and other potential pathogenic microorganisms, and a decrease in gram-positive (G+) bacteria such as Bifidobacterium and Lactobacillus 22. The considerable reduction of beneficial bacteria like Firmicutes and Actinobacteria within the intestine subsequent to chemotherapy unveils the pivotal role these bacteria assume in preserving the integrity of the intestinal barrier. In the future, safeguarding and restoring beneficial bacteria throughout chemotherapy and specifically regulating the quantity of Proteobacteria and Gram-negative bacteria constitute one of the research orientations for treating CID via the gut microbiome.

### Microbiota dysbiosis drives chemotherapy-induced diarrhea

In the one hand, in individuals undergoing fluoropyrimidine chemotherapy, only those experiencing diarrhea displayed a diminished α-diversity of their microbial community post-treatment. Specifically, this was manifested by a reduction in the abundance of Firmicutes and a corresponding increase in Bacteroidetes. Notably, these alterations were not evident in patients who did not develop diarrhea following chemotherapy 23. On the other hand, some studies have investigated the use of probiotics or manipulation of the ratio of organic acids in the gut as strategies to prevent or treat diarrhea by reducing chemotherapy-induced changes in the composition of the Intestinal Microbiota 24.

This suggests that chemotherapy-induced dysregulation of the intestinal microbiota may further impair the function of the intestinal mucosal barrier and promote the infiltration of harmful substances into the body, ultimately leading to a range of gastrointestinal problems, including diarrhea.

## Microbiota dysbiosis drives chemotherapy-induced diarrhea mechanisms

**Figure 1:**
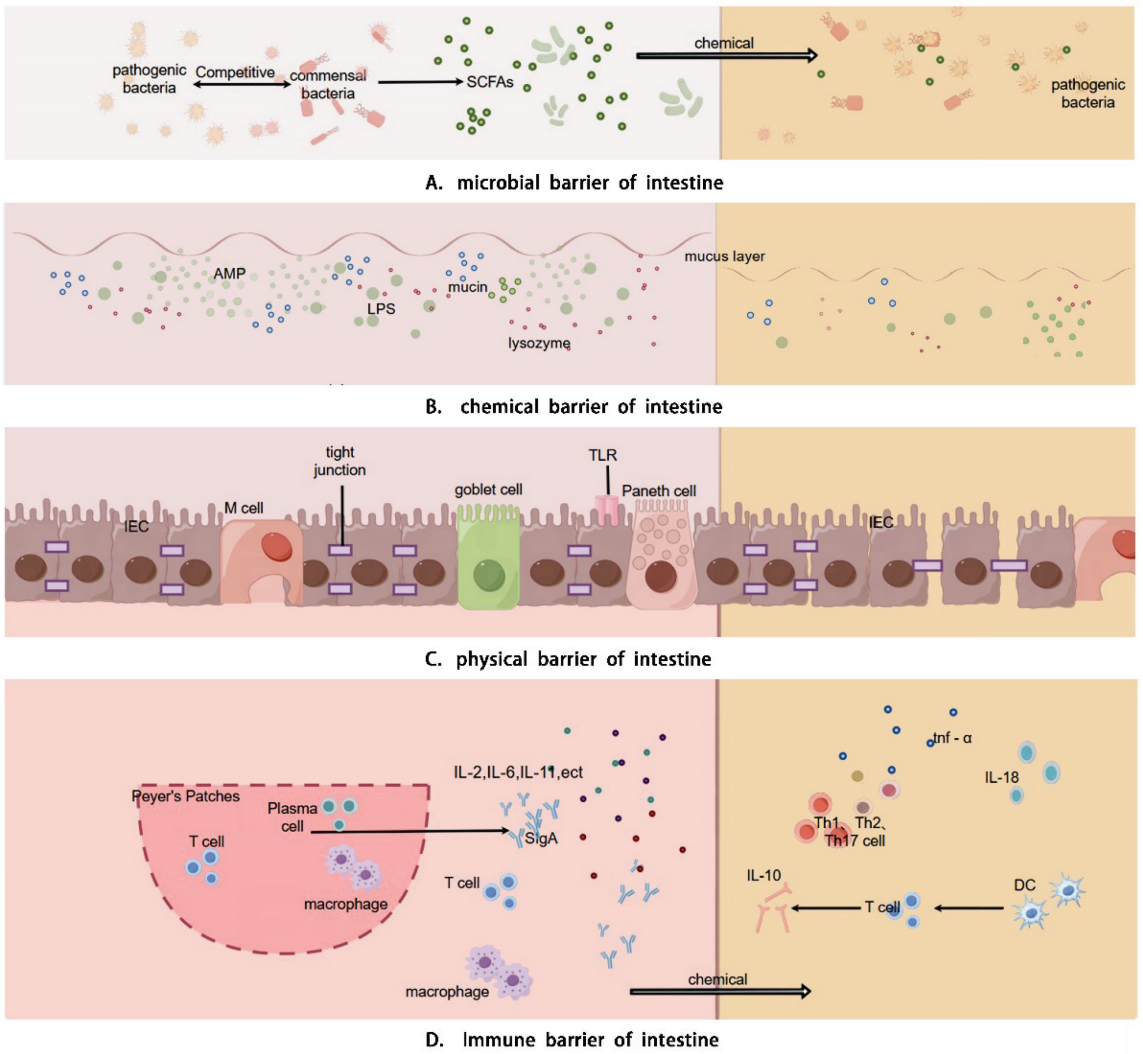
Physiological functions and mechanisms of the intestinal mucosal barrier. The intestinal barrier encompasses physical barrier, chemical barrier, immune barrier, and microbial barrier. The physical barrier is constituted by the intestinal absorptive cells, Paneth cells, and goblet cells that constitute the intestinal mucosal epithelium and the intercellular connections, which physically hinder microbial components from entering the intestine. The chemical barrier is primarily composed of the mucus layer consisting of digestive fluids, various digestive enzymes, lysozyme, bile acids, and mucins, which is capable of decomposing large molecular substances and bacterial components. The immune barrier is composed of the mucosal-associated lymphoid tissue (MALT) and diffuse immune cells, among which include Peyer's patches, follicular dendritic cells, and intraepithelial lymphocytes in the mucosa. It can recognize antigens, phagocytize viruses and intestinal pathogens, present antigens to immune cells, and generate humoral immunity and cellular immunity. The microbial barrier comprises the microorganisms and microbial metabolites residing in the intestine, and the commensal bacteria modulate the colonization of pathogenic bacteria by inhibiting their growth, consuming nutrients, generating bacteriocins, and influencing cell signaling pathways.

Intestinal barrier is comprised of physical, chemical, immune, and microbial components that interact to uphold intestinal health and restoration in the human body. Chemotherapy can induce diarrhea by detrimentally impacting these barriers through diverse mechanisms (Details in Fig. 1).

### Intestinal barriers and CID

CID is closely linked to gastrointestinal function and intestinal microbiota dysbiosis. Chemotherapy disrupts the balance of the gut microbiota, reducing beneficial bacteria and increasing harmful ones, which weakens the gut barrier and heightens permeability. This damage allows toxins and pathogens to enter, triggering inflammation and worsening diarrhea. Dysbiosis also alters metabolic functions, reducing the production of beneficial metabolites like short-chain fatty acids, further compromising gut health. Overall, microbiota dysbiosis plays a key role in the onset and progression of CID by impairing gut integrity and promoting inflammation.

#### Intestinal physical barrier disruption

The intestinal Physical barrier consists of absorptive cells, Paneth cells, intercellular connections, and goblet cells, forming the intestinal mucosal epithelial layer 25, 26. This barrier protects against pathogens, toxins, and allergens, while cellular connections prevent microbial invasion through paracellular pathways 27, 28. Intestinal microbiota and their byproducts, like SCFAs, promote epithelial cell proliferation, differentiation, and maturation, and reduce apoptosis, helping maintain barrier integrity. Intestinal commensal bacteria enhance epithelial tight junctions, reinforcing the gut's physical barrier 10, 29. Chemotherapy disrupts this balance by reducing Firmicutes and Actinobacteria, leading to decreased butyrate production 30. Butyrate supports tight junction assembly and expression via AMPK, GPR109A, and Akt pathways 31, 32. Impaired tight junctions increase epithelial permeability, causing mucositis and ulcers. Chemotherapy can directly damage the epithelium, leading to ulcers that intestinal bacteria can colonize, activating macrophages and increasing cytokine production, which exacerbates inflammation and leads to diarrhea 33.

#### Intestinal chemical barrier disruption

The chemical barrier consists of digestive juices, enzymes, lysozyme, bile acids, and mucus, which inhibit bacterial adhesion and colonization, degrade endotoxins, and destroy bacterial components 34-35. These components are regulated by intestinal microorganisms and their products 36. Mucin oligomerization forms a dense inner mucus layer that protects epithelial cells, while bacteria in the outer mucus layer maintain the barrier by: 1) degrading proteoglycans to produce SCFAs, supporting epithelial function 37, and 2) modulating goblet cell differentiation and mucin production 38, 39. Chemotherapy-induced malnutrition and reduced gastrointestinal hormone secretion impair protein and DNA metabolism, disrupting enzyme, gastric acid, and bile secretion, thus damaging the chemical barrier 40. Disrupted bile acid metabolism can reduce IL-10 production and increase mucosal permeability 41, while decreased antimicrobial molecules increase bacterial translocation 42. Probiotics may restore barrier function and reduce inflammation 43.

#### Intestinal immune barrier disruption

The immune barrier consists of gut-associated lymphoid tissue (GALT) and scattered immune cells. GALT, including Peyer's patches, lamina propria lymphocytes, and intraepithelial lymphocytes, recognizes antigens, phagocytoses pathogens, and facilitates immune responses 44, 45. The intestinal commensal microbiota not only includes probiotics but also includes some conditionally pathogenic bacteria, which together shape the intestinal immune function of the human body. Symbiotic intestinal bacteria influence the intestinal immune system, as evidenced by studies where healthy intestinal microbes restored immune functions in germ-free mice, improving CD4+ T cell differentiation and maintaining Treg and Th17 cell balance 46, 47. Segmented Filamentous Bacteria (SFB) enhance mucosal secretory IgA (SIgA) production and T cell differentiation 48.

Chemotherapy disrupts immune cell quantity and regulation, affecting both cellular and humoral immunity, and alters the CD4+/CD8+ T lymphocyte ratio. It reduces immune factor secretion (e.g., IL-2, IL-6, IL-11, SIgA) and increases pro-inflammatory factors (e.g., IL-1, TNF-alpha), impairing the intestinal immune barrier 49-51. This damage causes microbiota translocation and activates systemic inflammation through recognition of microbe-associated moflecular patterns (MAMPs) by innate immune cells 52. Toll-like receptors, STING pathways, and heat shock proteins play key roles in modulating these immunoinflammatory responses.

#### Intestinal microbiota regulation of key pathways in CID

**Figure 2:**
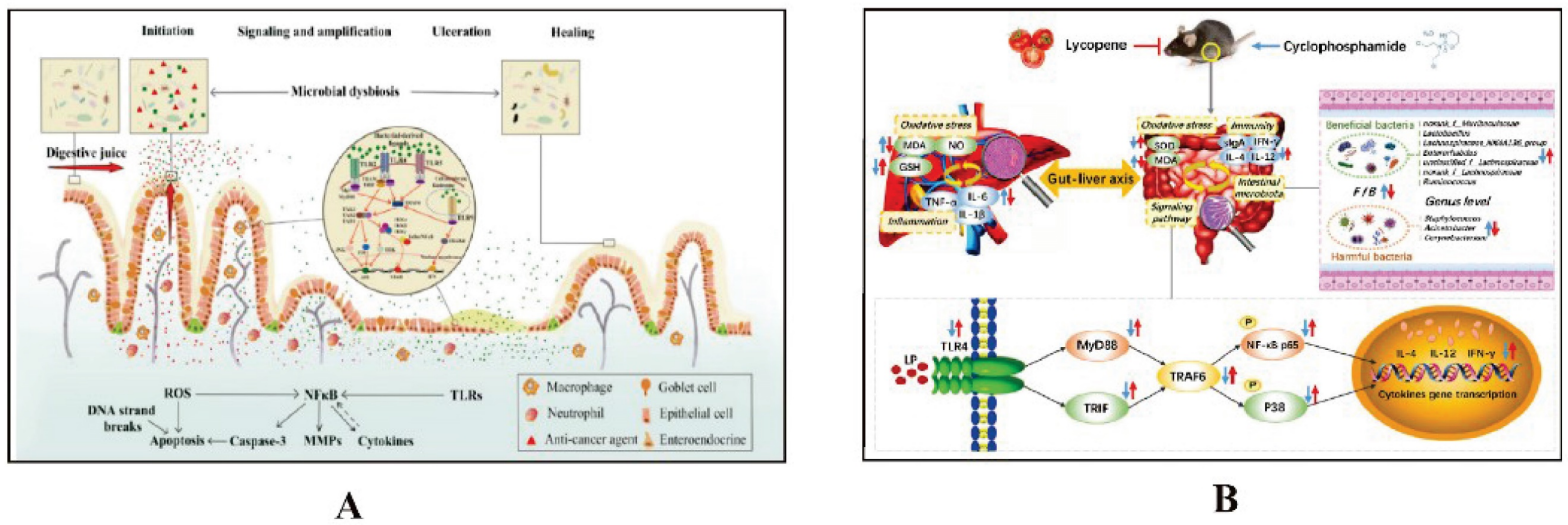
The mechanism by which intestinal microbiota regulates intestinal mucosal injury through NF-κB pathway. A. Impacts of chemotherapy-induced intestinal microbial dysbiosis on pathogenesis of mucositis and the non-intestinal manifestations of mucositis. Ref 22. Copyright © 2024 Elsevier GmbH. B. Preventive Mechanism of Intestinal Toxicity Caused by Cyclophosphamide Chemotherapy in Mice by Regulating TLR4-MyD88/TRIF-TRAF6 Signaling Pathway and Gut-Liver Axis. Ref 53. Copyright© 2021 by the authors.

**Figure 3:**
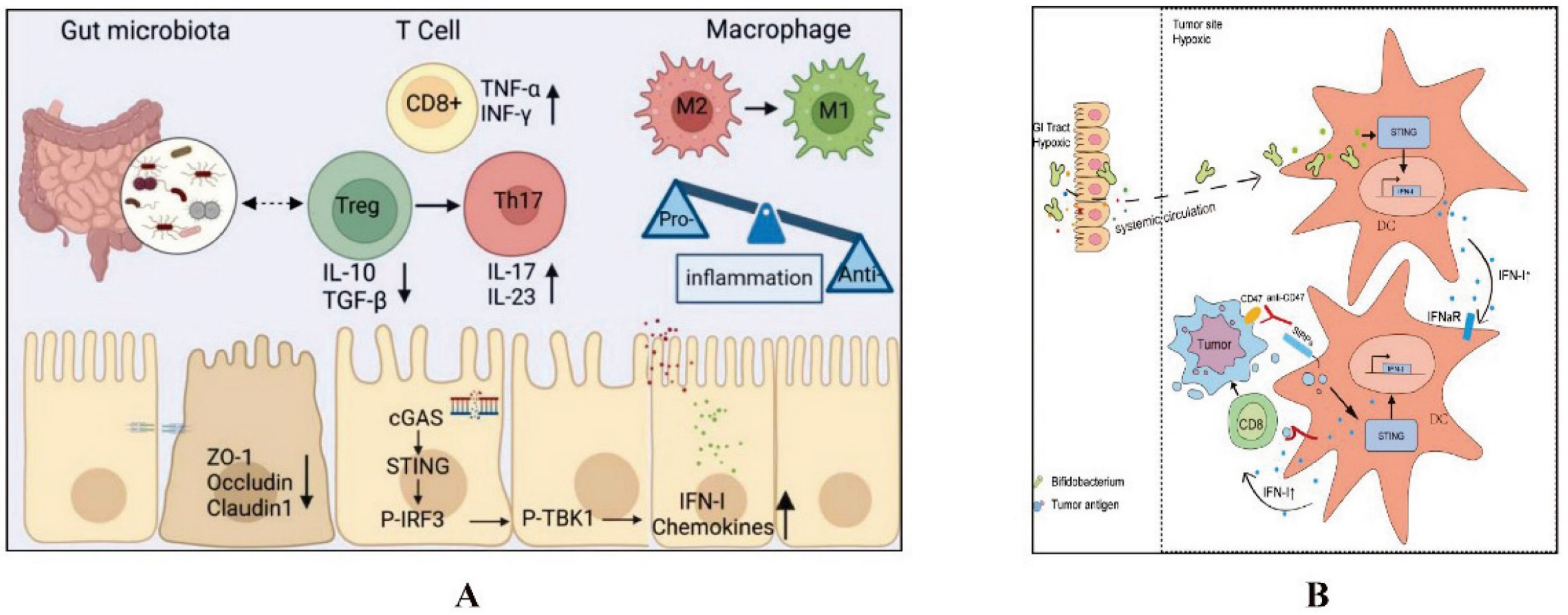
The mechanism by which intestinal microbiota regulates intestinal mucosal injury through STING pathways. A. Lactobacillus rhamnosus GG alleviates radiation-induced intestinal injury by modulating intestinal immunity and remodeling intestinal microbiota. Ref 54. Copyright © 2024 Elsevier GmbH. B. Intratumoral accumulation of gut microbiota facilitates CD47-based immunotherapy via STING signaling. Ref 55. ©Shi Y *et al.* Originally published in *JOURNAL NAME*. https://doi.org/10.1084/jem.20192282.

The intestinal microbiota regulates key immune pathways, including Toll-like receptors (TLRs) and the STING pathway, which play crucial roles in CID. Dysbiosis can activate these pathways excessively, leading to increased inflammation and intestinal barrier disruption. This exacerbates CID by enhancing intestinal permeability and inflammatory responses. Managing the microbiota can thus help mitigate the severity of CID (Details in Fig. 2 and Fig. 3).

#### Intestinal microbiota regulation of toll-like receptors (TLRs) in CID

Toll-like receptors (TLRs) play a pivotal role in preserving the integrity of the intestinal mucosal barrier by recognizing damage-associated molecular patterns (DAMPs) and microbe-associated molecular patterns (MAMPs). These receptors, expressed on both epithelial and immune cells within the gut, are critical for detecting pathogenic microbes and cellular stress signals. MAMPs originate from microbial components, including peptidoglycan and lipopolysaccharides, while DAMPs originate from host cells, including tumor cells, dead or dying cells. Upon activation by DAMPs or MAMPs, TLRs initiate downstream signaling cascades that enhance immune responses and fortify the mucosal barrier.

In conjunction with the enteric nervous system (ENS), TLRs influence intestinal motility, secretion, and immune regulation. The interaction between TLRs and ENS components, such as neurons and glial cells, helps to maintain mucosal homeostasis by regulating local immune responses and maintaining epithelial integrity. This coordinated response ensures that the intestinal mucosal barrier remains functional under physiological conditions, preventing pathogen infiltration and sustaining intestinal homeostasis. Consequently, TLR signaling within the DAMPs, MAMPs, and ENS pathways is essential for the maintenance of a resilient and adaptive intestinal mucosal barrier.

##### TLRs and damage-associated molecular patterns (DAMPs)

Toll-like receptors (TLRs) are a family of pattern-recognition receptors that activate relevant signaling pathways, such as the TLR/nuclear factor-κB (NF-κB) signaling pathway, by recognizing damage-associated molecular patterns (DAMPs) from stressed or dying cells, which result in the production of proinflammatory cytokines that mediate the inflammatory response 56, 57. Tollip, a negative regulator of TLR-mediated intrinsic immunity, exerts inhibitory effects on IRAK phosphorylation by binding to the TLR/MyD88/IL-1 receptor-activated kinase (IRAK) complex, thereby attenuating NF-κB transcription and suppressing inflammatory responses 58.

Under normal physiological conditions, persistent exposure of TLRs to TLR ligands, such as peptidoglycans, lipopolysaccharides (LPS), and nucleic acids from intestinal microbiota, induces an inflammatory response. However, the metabolites of commensal bacteria - specifically butyrate salts - have been found to enhance the expression of TOLLIP in intestinal epithelial cells and inhibit inflammation. This regulatory mechanism maintains a dynamic balance between pro-inflammatory and anti-inflammatory responses by ensuring high levels of TOLLIP and low levels of TLRs in the gut, thereby keeping the body in a state of low inflammation 59.

The administration of chemotherapeutic drugs promotes the development of gastrointestinal mucositis by compromising the integrity of the intestinal immune barrier and downregulating TOLLIP expression, resulting in excessive activation of TLRs and subsequent activation of the NF-κB signaling pathway.

##### TLRs and microbial-associated molecular patterns (MAMPs)

**Figure 4:**
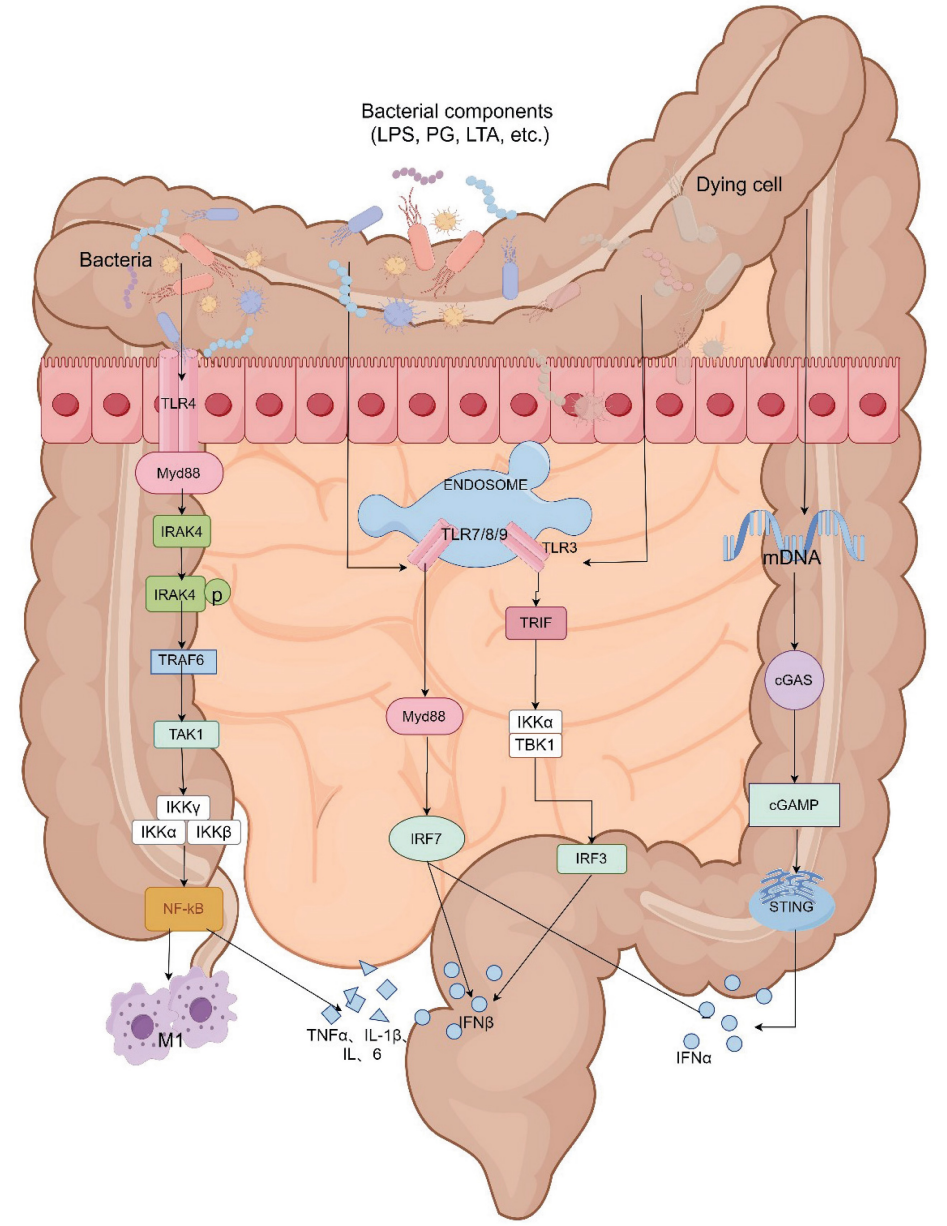
Intestinal microbiota mediate CID through the TLR receptor and cGAS-STING pathway. The TLR4 is located on the cell surface. TLR3, TLR7/8, and TLR9 are present in a multiprotein signaling complex called the "endosome." Except for TLR3, all other TLRs recruit Myeloid Differentiation Primary Response 88 (MyD88) as an adaptor protein. MyD88 attracts IL-1 Receptor-Associated Kinase 4 (IRAK-4) to the TLRs, which then undergo phosphorylation and activate IRAK-1. After interacting with TRAF6, IRAK-1 activates the IKK complex and leads to NF-kB activation. Both TLR3 and TLR4 recruit Toll/IL-1 Receptor Domain Inducing IFN-beta (TRIF) protein. The signaling pathway of both TLRs results in NF-kB activation and/or expression of type I interferons (IFNs).

In addition, dysbiosis of the intestinal microbiota reduces tight junction expression, increases epithelial permeability, decreases secretion of antimicrobial substances, weakens antibacterial ability. Chemotherapeutic drugs and their metabolites may penetrate the intestinal mucosa to kill commensal bacteria, promote growth of pathogenic bacteria, erosion, ulceration and death of intestinal cells can lead to unrestricted entry of bacterial and microbial-associated molecular patterns (MAMPs) including peptidoglycans, LPS, and lipophosphatidic wall acids, on various microorganisms into the lamina propria layer. ST4 is a major adapter protein in the TLR signaling pathway. MAMPs are directly recognized by Toll-like receptors (TLRs), mediated inflammation through activation of MyD88-dependent pathway (except for TLR3 signaling activation) and MyD88-independent TRIF/TRAM pathway (TLR3 and some TLR4 signals) 60. Activation of the MyD88-dependent pathway leads to the activation of the NF-κB pathway, which promotes the synthesis of pro-inflammatory cytokines and mediates inflammatory responses. Activation of the MyD88-independent pathway results in secretion of IFN-β 61, and most TLR signaling acts through the Myd88-dependent pathway. Meanwhile, the activation of the NF-κB signaling pathway is closely linked to M1 macrophage polarization. Upon stimulation by LPS, M1 macrophages secrete pro-inflammatory cytokines such as interleukin IL-6, IL-1β, TNF-α, and iNOS 62, thereby exacerbating inflammatory damage and subsequently leading to the development of diarrhea (Details in Fig. 4).

##### TLRs and the Enteric Nervous System pathway (ENS)

ENS is the primary regulator of gastrointestinal function, responsible for controlling intestinal functions such as absorption, secretion, motility, and vascular tone 63. Moreover, the microbiota and microbial factors (such as SCFAs and LPS) can promote the maturation of ENS function, which in turn coordinates the response of intestinal microbiota and transmits it throughout the entire intestine 64, 65.

TLR4 is expressed in both mouse neurons and glial cells, enabling intestinal neurons and glial cells to directly sense gut bacteria and activate gastrointestinal neural responses, thereby regulating intestinal motility 66, 67. Chemotherapy induces damage to the intestinal barrier function, leading to changes in the gut microbiota, increased expression of TLR4, immune activation, and low-grade inflammation in the intestines. These alterations can affect the structure and function of intestinal neurons, resulting in impaired gastrointestinal motility and subsequent development of functional gastrointestinal disorders that ultimately lead to diarrhea 68.

Composition of gut microbiota, leading to impaired intestinal barrier function and repair pathways, disruption of intestinal integrity, and damage to the enteric nervous system. Pathogenic bacteria can directly stimulate intestinal epithelial nerves through TLR, activating the enteric nervous system immune pathway 69. By regulating multiple neuronal circuits and sensory neurons in the local intestine, a large number of neuropeptides such as vasoactive intestinal peptide (VIP), substance P, and calcitonin gene-related peptide (CGRP) are produced, resulting in neurogenic inflammation 70. enteric glial cells (EGCs) are an essential component of the ENS and are also considered antigen-presenting cells. Upon activation of the ENS, they can produce inflammatory factors such as TNF-α, IL-1β, and IL-6, leading to the activation of macrophages, mast cells, and T cells 71, further exacerbating intestinal inflammation and causing diarrhea.

Gram-negative bacteria and a small number of Gram-positive bacteria in the intestines can be recognized by TLRs, mainly TLR2, TLR4, TLR5, and TLR9. They are then transported into cells for processing, which activates intracellular NOD-like receptors (NLRs) and promotes the formation of inflammasomes through CARD-CARD interactions. This subsequently triggers diarrhea through downstream NF-κB and MAPK-mediated inflammation 72.

#### STING pathway

##### Intestinal Microbiota and STING pathway modulation

Chemotherapy disrupts the intestinal microbiota, leading to dysbiosis and compromised intestinal mucosal barrier function. This disruption results in an increased production of microbial metabolites and antigens, which can exacerbate inflammatory responses. The STING (stimulator of interferon genes) pathway, critical for regulating innate immune responses, is activated by cytosolic DNA from damaged cells or microbial sources. STING is a protein located in the endoplasmic reticulum that can be activated and initiate type I interferon (IFN-I) response when stimulated by DNA. Mice with STING knockout exhibit elongated colonic villi, shallower crypts, and a shift towards pro-inflammatory microbiota in the intestinal tract 73, demonstrating the crucial role of STING in regulating and maintaining intestinal homeostasis. In the healthy intestine, the expression level of STING is low. The intrinsic STING pathway maintains intestinal barrier function by stimulating the secretion of IFN-I and interferon-stimulated genes (ISGs), enhancing the integrity and regeneration of the epithelial barrier, promoting the production of antimicrobial peptides by PAN cells, and synthesizing mucus by cuprocytes, among many other mechanisms 74.

##### Activation of the STING pathway

In a normal physiological state, STING is activated in immune cells, such as macrophages and dendritic cells, by cytosolic DNA. Chemotherapy-induced cellular stress and intestinal damage elevate the release of mitochondrial and microbial DNA. This DNA is recognized by the cyclic GMP-AMP synthase (cGAS) enzyme, which generates cGAMP and subsequently activates STING. Activated STING triggers the production of type I interferons and other pro-inflammatory cytokines.

##### Regulation of STING by the intestinal microbiota

The intestinal microbiota plays a significant role in modulating the STING pathway. Beneficial microbial species help maintain the integrity of the intestinal barrier, reducing the translocation of microbial DNA into the bloodstream and preventing excessive STING activation. Additionally, bacterial metabolites such as SCFAs influence immune responses by modulating cytokine production and limiting STING activation, thereby preventing excessive inflammation.

##### Impact on chemotherapy-induced inflammation and diarrhea

A balanced intestinal microbiota helps regulate the STING pathway, thereby controlling inflammatory signaling during chemotherapy. Effective modulation of STING by the microbiota can prevent excessive inflammation, reducing mucosal damage and intestinal permeability, which are key factors in chemotherapy-induced diarrhea. By supporting immune responses and intestinal repair, the microbiota plays a crucial role in mitigating the severity of diarrhea and promoting mucosal healing post-chemotherapy.

Meanwhile, TOLLIP can promote the autophagic-lysosomal degradation of STING, thereby blocking the phosphorylation of downstream signaling molecules TBK1, IRF3, and IκBα, reducing the production of IFN-I 75. The toxic damage caused by chemotherapy affects intestinal epithelial cells, leading to bacterial translocation. Microbial DNA released by invading pathogens into the cytoplasm is recognized by the cytoplasmic DNA sensor cyclic GMP-AMP synthase (cGAS), which generates cGAMP and activates STING. Decreased expression of TOLLIP can reduce endogenous STING degradation, while bacterial products induce ubiquitination of STING in myeloid cells, resulting in its aggregation in intestinal macrophages and monocytes. This induces the expression of IFN-I and various other inflammatory cytokines, ultimately causing intestinal inflammation and diarrhea 76-78.

#### Heat shock proteins

Heat shock protein (HSPs70) is located inside cells and exerts anti-inflammatory effects by inhibiting the production and release of various inflammatory factors, such as TNF-α, IL-1, IL-6 in monocyte macrophages 79. Haoyu L observed mice with colitis and found that the use of L. reuteri was able to reduce the level of inflammation in their mice. Colitis decreased the expression of heat shock proteins HSP70 and HSP25, while L. reuteri treatment increased the expression of these proteins. In addition, the changes of HSPs were found to be correlated to bacterial load and epithelial cell proliferation 80. However, the specific mechanism by which gut dysbiosis affects HSP70 remains unclear.

## Clinical applications and potential of intestinal microbiota regulation in treating CID

Currently, treatments for chemotherapy-induced diarrhea (CID) include Loperamide, Octreotide, DTO, and Crofelemer. Loperamide is effective for mild to moderate CID but has limited efficacy in severe cases and may cause adverse effects like intestinal paralysis and arrhythmias 81. Octreotide is effective for severe CID but is expensive 82. DTO has a historical role but lacks strong contemporary evidence for CID treatment 83. Crofelemer shows potential but needs further validation due to limited clinical trials 84. Overall, these treatments face challenges such as restricted effectiveness, high costs, and significant side effects, underscoring the need for more effective and safer options. Intestinal microbiota regulation in Treating CID shows great potential.

### Probiotics

Probiotics offer a promising approach for managing chemotherapy-induced diarrhea (CID) by improving intestinal microbiota balance, protecting the epithelial barrier, and reducing intestinal permeability 85, 86. They restore gut microbiota after chemotherapy, release antimicrobials, and mitigate mucosal inflammation, which collectively help reduce CID 87, 88. Probiotic therapy involves administering live probiotics to support gastrointestinal health. Common probiotic strains include Bifidobacterium spp. and Lactobacillus spp., available in both single-strain and mixed-strain supplements 89 (Showen in table 1).

Studies demonstrate the efficacy of probiotics in CID management. For instance, S. thermophilus (ST4) significantly reduced diarrhea scores in mice with enteritis, by maintaining epithelial integrity and decreasing inflammatory cytokines 90. In lung cancer patients, Clostridium butyricum reduced diarrhea incidence compared to a placebo and improved nausea and vomiting symptoms, while also lowering systemic inflammation indicators 89.

Meta-analyses show that probiotic supplementation significantly decreases CID incidence and severity, with varying effects across different probiotic formulations. Studies have reported mixed results, such as reduced diarrhea incidence with Bacteroides fragilis 839 91, and C. butyricum's effectiveness in preventing CID 89.

Lactobacillus rhamnosus GG showed promising results, though further research with larger samples is needed 92. Mixed probiotic formulations also show potential, though effectiveness may vary by strain combination 93, 94.

While probiotics can reduce CID, their safety must be considered. They may cause adverse reactions in a small percentage of individuals and potentially increase infection risk in vulnerable populations, though such risks are generally low 95, 96. More extensive studies are needed to evaluate the impact of probiotics across different demographics and cancer types, and to optimize treatment strategies by exploring mechanisms, dose-response relationships, and strain interactions.

### Fecal microbiota transplantation

Fecal microbiota transplantation (FMT) holds promise for alleviating chemotherapy-induced diarrhea (CID) by restoring intestinal microbiota balance, enhancing mucosal barrier function, and modulating immune responses 99. Traditionally, FMT involved endoscopic or nasojejunal delivery of fecal samples, but now it is commonly administered via encapsulated oral capsules 100, 101. Studies indicate FMT's effectiveness: in mice, FMT reduced diarrhea severity and improved gut morphology and inflammation 102. In human trials, FMT also decreased diarrhea incidence and inflammatory markers while improving microbial community function 101.

FMT improves CID through several mechanisms, including restoring microbiota balance, reducing bacterial translocation, and enhancing intestinal barrier integrity. It can reverse chemotherapy-induced damage and boost beneficial bacteria like Bifidobacterium and Lactobacillus, improving the intestinal environment 101, 102. However, FMT's success depends on donor microbiome quality and matching with the recipient, and it involves complex procedures with potential risks. Adverse events, though mostly mild, can include gastrointestinal discomfort, and rare severe outcomes like infections or disease recurrence 103, 104.

Intestinal microbiota regulation offers promising potential for the future of treating CID. Approaches such as probiotics and fecal microbiota transplantation (FMT) not only hold promise for improving intestinal barrier function and reducing inflammation but also present advantages such as minimal side effects compared to conventional treatments. These methods can help restore gut microbiota balance with fewer adverse reactions, making them a compelling option for CID management. Continued research and development are likely to enhance their efficacy and application, positioning them as valuable tools in future therapeutic strategies for CID.

## Conclusion

In the intestinal microenvironment, the intestinal microbiota plays a pivotal role in maintaining the integrity of the intestinal barrier function. Imbalances in the intestinal microbiota, along with disruptions in the intestinal barrier and inflammation of the intestinal mucosa, are significant mechanisms underlying CID. Disruption of the intestinal microbiome can stimulate TLR receptors through the DAMPS, MAMPS, or ENS pathways to activate the inflammation pathway, or activate the cGAS-STING pathway to cause excessive inflammation. However, current treatment options for CID have limitations as they fail to restore normal gut microbiota. Therefore, modulation of gut microbiota composition can effectively reduce both the occurrence and severity of chemotherapy-induced diarrhea while improving treatment efficacy and overall quality of life. It has been proven that the use of probiotics or FMT for chemotherapy-induced diarrhea is effective, but whether probiotics reduce the incidence of CID by inhibiting TLRs and STING pathways in the intestinal tract still needs further research. Meanwhile complex microbial compositions and individual variations within gut microbiota, the specific mechanism through which the strain exerts its therapeutic impacts has not yet been discovered. Moreover, the existing probiotic preparations are still limited compared to the diversity of gut microbiota, and FMT is less convenient than oral medication. Furthermore, there is currently a lack of large-scale studies that have achieved higher levels of evidence grading and obtained guideline recommendations for the diagnosis and treatment of chemotherapy-induced diarrhea. Consequently, future directions should focus on conducting relevant basic research aimed at analyzing key pathways involved while developing more targeted.

## Figures and Tables

**Table 1:** The clinical research on the effects of probiotic strains on CID over the past decade.

Age	Author	Probiotics (Species, Components)	(Percentage of Patients,%)	Study group	Control group
2024	Ting Z 91	Bacteroides fragilis 839	Incidence of Diarrhea	12 (15%)	24 (30%)
2023	Wei H 93	compound probiotic preparation	Incidence of Diarrhea	3 (7.14%)	21 (42.86%)
2023	Eghbali A 97	LactoCare synbiotic	Modeling odds of diarrhea (OR)	1.45 (1.17-4.01)	2.51 (1.35-4.13)
2019	Tian Y 89	C. butyricum	Incidence of no diarrhea	15 (75%)	8 (38.09%)
2019	Zaharuddin L 98	mixture of six viable strains(Lactobacillus and Bifidobacterium)	Incidence of no diarrhea	2 (25%)	1 (16.67%)
2019	Reyna-Figueroa J 92	Lactobacillus rhamnosus GG. A	Incidence of Diarrhea	0 (zero)	3 (10%)
2015	Mego M 94	mixture of ten viable strains(Bifidobacterium, Lactobacillus , Streptococcus thermopilus and so on)	Incidence of no diarrhea	14 (60.9%)	9 (39.1%)

## Abbreviations

CID: Chemotherapy-induced diarrhea
SCFA: short-chain fatty acid
AMP: anti-microbial peptide
LPS: Lipopolysaccharides
IEC: Intestinal epithelial cells
TLR: Toll-like receptors
DC: Dendritic cell
GALT: gut-associated lymphoid tissue
secretory IgA: SIgA
SFB: Segmented Filamentous Bacteria
MAMPs: microbe-associated molecular patterns
DAMPs: damage-associated molecular patterns
ENS: enteric nervous system
NF-κB: nuclear factor-κB
VIP: vasoactive intestinal peptide
EGCs: enteric glial cells
NLRs: NOD-like receptors
STING: stimulator of interferon genes
ISGs: interferon-stimulated genes
FMT: Fecal microbiota transplantation

## References

[1] Andreyev J, Ross P, Donnellan C, Lennan E, Leonard P, Waters C (2014). Guidance on the management of diarrhoea during cancer chemotherapy. Lancet Oncol.

[2] Thet D, Areepium N, Siritientong T (2023). Effects of Probiotics on Chemotherapy-induced Diarrhea. Nutr Cancer.

[3] Benson AB, Ajani JA, Catalano RB, Engelking C, Kornblau SM, Martenson JA (2004). Recommended Guidelines for the Treatment of Cancer Treatment-Induced Diarrhea. Journal of Clinical Oncology.

[4] McQuade RM, Stojanovska V, Abalo R, Bornstein JC, Nurgali K (2016). Chemotherapy-Induced Constipation and Diarrhea: Pathophysiology, Current and Emerging Treatments. Front Pharmacol.

[5] Maroun JA, Anthony LB, Blais N, Burkes R, Dowden SD, Dranitsaris G (2007). Prevention and management of chemotherapy-induced diarrhea in patients with colorectal cancer: a consensus statement by the Canadian Working Group on Chemotherapy-Induced Diarrhea. Curr Oncol.

[6] Dahlgren D, Lennernäs H (2023). Review on the effect of chemotherapy on the intestinal barrier: Epithelial permeability, mucus and bacterial translocation. Biomed Pharmacother.

[7] Yu Q-Q, Zhang H, Guo Y, Han B, Jiang P (2022). The Intestinal Redox System and Its Significance in Chemotherapy-Induced Intestinal Mucositis. Oxidative Medicine and Cellular Longevity.

[8] Lin L, Zhang J (2017). Role of intestinal microbiota and metabolites on gut homeostasis and human diseases. BMC Immunology.

[9] Wang Y, Wang M, Chen J, Li Y, Kuang Z, Dende C (2023). The gut microbiota reprograms intestinal lipid metabolism through long noncoding RNA Snhg9. Science.

[10] Kayama H, Okumura R, Takeda K (2020). Interaction Between the Microbiota, Epithelia, and Immune Cells in the Intestine. Annu Rev Immunol.

[11] Van Vliet MJ, Harmsen HJM, De Bont ESJM, Tissing WJE (2010). The Role of Intestinal Microbiota in the Development and Severity of Chemotherapy-Induced Mucositis. PLoS Pathogens.

[12] Wang Z, Zhou Y, Luo A, Heng X, Liu J, Wang H (2023). Lactobacillus salivarius CPU-01 Ameliorates Temozolomide-Induced Intestinal Mucositis by Modulating Gut Microbiota, Maintaining Intestinal Barrier, and Blocking Pro-inflammatory Cytokines. Probiotics and Antimicrobial Proteins.

[13] Souza RO, Miranda VC, Quintanilha MF, Gallotti B, Oliveira SRM, Silva JL (2024). Evaluation of the Treatment with Akkermansia muciniphila BAA-835 of Chemotherapy-induced Mucositis in Mice. Probiotics and Antimicrobial Proteins.

[14] Batista VL, De Jesus LCL, Tavares LM, Barroso FLA, Fernandes LJDS, Freitas ADS (2022). Paraprobiotics and Postbiotics of Lactobacillus delbrueckii CIDCA 133 Mitigate 5-FU-Induced Intestinal Inflammation. Microorganisms.

[15] Li G, Lin J, Zhang C, Gao H, Lu H, Gao X (2021). Microbiota metabolite butyrate constrains neutrophil functions and ameliorates mucosal inflammation in inflammatory bowel disease. Gut Microbes.

[16] Hugon P, Dufour J-C, Colson P, Fournier P-E, Sallah K, Raoult D (2015). A comprehensive repertoire of prokaryotic species identified in human beings. The Lancet Infectious Diseases.

[17] Meta HITC, Li J, Jia H, Cai X, Zhong H, Feng Q (2014). An integrated catalog of reference genes in the human gut microbiome. Nature Biotechnology.

[18] Meta HITC, Arumugam M, Raes J, Pelletier E, Le Paslier D, Yamada T (2011). Enterotypes of the human gut microbiome. Nature.

[19] Montassier E, Gastinne T, Vangay P, Al-Ghalith GA, Bruley Des Varannes S, Massart S (2015). Chemotherapy-driven dysbiosis in the intestinal microbiome. Alimentary Pharmacology & Therapeutics.

[20] Viaud S, Saccheri F, Mignot G, Yamazaki T, Daillère R, Hannani D (2013). The Intestinal Microbiota Modulates the Anticancer Immune Effects of Cyclophosphamide. Science.

[21] Fijlstra M, Ferdous M, Koning AM, Rings EHHM, Harmsen HJM, Tissing WJE (2015). Substantial decreases in the number and diversity of microbiota during chemotherapy-induced gastrointestinal mucositis in a rat model. Supportive Care in Cancer.

[22] Wei L, Wen X-S, Xian CJ (2021). Chemotherapy-Induced Intestinal Microbiota Dysbiosis Impairs Mucosal Homeostasis by Modulating Toll-like Receptor Signaling Pathways. International Journal of Molecular Sciences.

[23] Kawasaki Y, Kakimoto K, Tanaka Y, Shimizu H, Nishida K, Numa K (2023). Relationship between Chemotherapy-Induced Diarrhea and Intestinal Microbiome Composition. Digestion.

[24] Kaliannan K, Donnell SO, Murphy K, Stanton C, Kang C, Wang B (2022). Decreased Tissue Omega-6/Omega-3 Fatty Acid Ratio Prevents Chemotherapy-Induced Gastrointestinal Toxicity Associated with Alterations of Gut Microbiome. International Journal of Molecular Sciences.

[25] Peterson LW, Artis D (2014). Intestinal epithelial cells: regulators of barrier function and immune homeostasis. Nature Reviews Immunology.

[26] Pelaseyed T, Bergström JH, Gustafsson JK, Ermund A, Birchenough GMH, Schütte A (2014). The mucus and mucins of the goblet cells and enterocytes provide the first defense line of the gastrointestinal tract and interact with the immune system. Immunological Reviews.

[27] Usuda H, Okamoto T, Wada K (2021). Leaky Gut: Effect of Dietary Fiber and Fats on Microbiome and Intestinal Barrier. International Journal of Molecular Sciences.

[28] Zihni C, Mills C, Matter K, Balda MS (2016). Tight junctions: from simple barriers to multifunctional molecular gates. Nature Reviews Molecular Cell Biology.

[29] Allam-Ndoul B, Castonguay-Paradis S, Veilleux A (2020). Gut Microbiota and Intestinal Trans-Epithelial Permeability. International Journal of Molecular Sciences.

[30] Al-Sadi R, Dharmaprakash V, Nighot P, Guo S, Nighot M, Do T (2021). Bifidobacterium bifidum Enhances the Intestinal Epithelial Tight Junction Barrier and Protects against Intestinal Inflammation by Targeting the Toll-like Receptor-2 Pathway in an NF-κB-Independent Manner. International Journal of Molecular Sciences.

[31] Li X, Wang C, Zhu J, Lin Q, Yu M, Wen J (2022). Sodium Butyrate Ameliorates Oxidative Stress-Induced Intestinal Epithelium Barrier Injury and Mitochondrial Damage through AMPK-Mitophagy Pathway. Oxidative Medicine and Cellular Longevity.

[32] Chen G, Ran X, Li B, Li Y, He D, Huang B (2018). Sodium Butyrate Inhibits Inflammation and Maintains Epithelium Barrier Integrity in a TNBS-induced Inflammatory Bowel Disease Mice Model. EBioMedicine.

[33] Villa A, Sonis ST (2015). Mucositis: pathobiology and management. Curr Opin Oncol.

[34] Louis P, Flint HJ (2009). Diversity, metabolism and microbial ecology of butyrate-producing bacteria from the human large intestine. FEMS Microbiology Letters.

[35] Ragland SA, Criss AK (2017). From bacterial killing to immune modulation: Recent insights into the functions of lysozyme. PLOS Pathogens.

[36] Jakobsson HE, Rodríguez-Piñeiro AM, Schütte A, Ermund A, Boysen P, Bemark M (2015). The composition of the gut microbiota shapes the colon mucus barrier. EMBO reports.

[37] Hansson GC (2020). Mucins and the Microbiome. Annual Review of Biochemistry.

[38] Wrzosek L, Miquel S, Noordine M-L, Bouet S, Chevalier-Curt MJ, Robert V (2013). Bacteroides thetaiotaomicron and Faecalibacterium prausnitziiinfluence the production of mucus glycans and the development of goblet cells in the colonic epithelium of a gnotobiotic model rodent. BMC Biology.

[39] Shin N-R, Lee J-C, Lee H-Y, Kim M-S, Whon TW, Lee M-S (2014). An increase in the Akkermansia spp. population induced by metformin treatment improves glucose homeostasis in diet-induced obese mice. Gut.

[40] Vanhoecke B, Bateman E, Mayo B, Vanlancker E, Stringer A, Thorpe D (2015). Dark Agouti rat model of chemotherapy-induced mucositis: Establishment and current state of the art. Experimental Biology and Medicine.

[41] Fang Z-Z, Zhang D, Cao Y-F, Xie C, Lu D, Sun D-X (2016). Irinotecan (CPT-11)-induced elevation of bile acids potentiates suppression of IL-10 expression. Toxicology and Applied Pharmacology.

[42] Da Silva Ferreira AR, Wardill HR, Tissing WJE, Harmsen HJM (2020). Pitfalls and novel experimental approaches to optimize microbial interventions for chemotherapy-induced gastrointestinal mucositis. Curr Opin Support Palliat Care.

[43] Jingjing F, Weilin J, Shaochen S, Aman K, Ying W, Yanyi C (2024). A Probiotic Targets Bile Acids Metabolism to Alleviate Ulcerative Colitis by Reducing Conjugated Bile Acids. Mol Nutr Food Res.

[44] Kobayashi N, Takahashi D, Takano S, Kimura S, Hase K (2019). The Roles of Peyer's Patches and Microfold Cells in the Gut Immune System: Relevance to Autoimmune Diseases. Front Immunol.

[45] Ohno H (2016). Intestinal M cells. J Biochem.

[46] Pollard M, Sharon N (1970). Responses of the Peyer's Patches in Germ-Free Mice to Antigenic Stimulation. Infect Immun.

[47] Bouskra D, Brezillon C, Berard M, Werts C, Varona R, Boneca IG (2008). Lymphoid tissue genesis induced by commensals through NOD1 regulates intestinal homeostasis. Nature.

[48] Lécuyer E, Rakotobe S, Lengliné-Garnier H, Lebreton C, Picard M, Juste C (2014). Segmented Filamentous Bacterium Uses Secondary and Tertiary Lymphoid Tissues to Induce Gut IgA and Specific T Helper 17 Cell Responses. Immunity.

[49] Li C, Duan S, Li Y, Pan X, Han L (2021). Polysaccharides in natural products that repair the damage to intestinal mucosa caused by cyclophosphamide and their mechanisms: A review. Carbohydrate Polymers.

[50] Sougiannis AT, VanderVeen BN, Enos RT, Velazquez KT, Bader JE, Carson M (2019). Impact of 5 fluorouracil chemotherapy on gut inflammation, functional parameters, and gut microbiota. Brain, Behavior, and Immunity.

[51] Akbarali HI, Muchhala KH, Jessup DK, Cheatham S (2022). Chemotherapy induced gastrointestinal toxicities. Adv Cancer Res.

[52] Akira S, Uematsu S, Takeuchi O (2006). Pathogen Recognition and Innate Immunity. Cell.

[53] Pan X, Niu X, Li Y, Yao Y, Han L (2022). Preventive Mechanism of Lycopene on Intestinal Toxicity Caused by Cyclophosphamide Chemotherapy in Mice by Regulating TLR4-MyD88/TRIF-TRAF6 Signaling Pathway and Gut-Liver Axis. Nutrients.

[54] Ll Z, Jy X, Y X, P W, Yw J, W W (2024). Lactobacillus rhamnosus GG alleviates radiation-induced intestinal injury by modulating intestinal immunity and remodeling gut microbiota. Microbiological research.

[55] Shi Y, Zheng W, Yang K, Harris KG, Ni K, Xue L (2020). Intratumoral accumulation of gut microbiota facilitates CD47-based immunotherapy via STING signaling. Journal of Experimental Medicine.

[56] Duan T, Du Y, Xing C, Wang HY, Wang R-F (2022). Toll-Like Receptor Signaling and Its Role in Cell-Mediated Immunity. Frontiers in Immunology.

[57] Vidya MK, Kumar VG, Sejian V, Bagath M, Krishnan G, Bhatta R (2018). Toll-like receptors: Significance, ligands, signaling pathways, and functions in mammals. International Reviews of Immunology.

[58] Capelluto DGS (2012). Tollip: a multitasking protein in innate immunity and protein trafficking. Microbes and Infection.

[59] Kelly D, Campbell JI, King TP, Grant G, Jansson EA, Coutts AGP (2004). Commensal anaerobic gut bacteria attenuate inflammation by regulating nuclear-cytoplasmic shuttling of PPAR-γ and RelA. Nature Immunology.

[60] Liu C, Han C, Liu J (2019). The Role of Toll-Like Receptors in Oncotherapy. Oncology Research Featuring Preclinical and Clinical Cancer Therapeutics.

[61] Yang Y, Feng R, Wang Y-Z, Sun H-W, Zou Q-M, Li H-B (2020). Toll-like receptors: Triggers of regulated cell death and promising targets for cancer therapy. Immunology Letters.

[62] Liu X, Ren X, Zhou L, Liu K, Deng L, Qing Q (2022). Tollip Orchestrates Macrophage Polarization to Alleviate Intestinal Mucosal Inflammation. Journal of Crohn's and Colitis.

[63] Seguella L, Gulbransen BD (2021). Enteric glial biology, intercellular signalling and roles in gastrointestinal disease. Nature Reviews Gastroenterology & Hepatology.

[64] Holland AM, Bon-Frauches AC, Keszthelyi D, Melotte V, Boesmans W (2021). The enteric nervous system in gastrointestinal disease etiology. Cellular and Molecular Life Sciences.

[65] Vicentini FA, Keenan CM, Wallace LE, Woods C, Cavin J-B, Flockton AR (2021). Intestinal microbiota shapes gut physiology and regulates enteric neurons and glia. Microbiome.

[66] Anitha M, Vijay-Kumar M, Sitaraman SV, Gewirtz AT, Srinivasan S (2012). Gut Microbial Products Regulate Murine Gastrointestinal Motility via Toll-Like Receptor 4 Signaling. Gastroenterology.

[67] Barajon I, Serrao G, Arnaboldi F, Opizzi E, Ripamonti G, Balsari A (2009). Toll-like Receptors 3, 4, and 7 Are Expressed in the Enteric Nervous System and Dorsal Root Ganglia. Journal of Histochemistry & Cytochemistry.

[68] Hao MM, Foong JPP, Bornstein JC, Li ZL, Vanden Berghe P, Boesmans W (2016). Enteric nervous system assembly: Functional integration within the developing gut. Developmental Biology.

[69] Bajic JE, Johnston IN, Howarth GS, Hutchinson MR (2018). From the Bottom-Up: Chemotherapy and Gut-Brain Axis Dysregulation. Frontiers in Behavioral Neuroscience.

[70] Chavan SS, Pavlov VA, Tracey KJ (2017). Mechanisms and Therapeutic Relevance of Neuro-immune Communication. Immunity.

[71] Li H, Fan C, Lu H, Feng C, He P, Yang X (2020). Protective role of berberine on ulcerative colitis through modulating enteric glial cells-intestinal epithelial cells-immune cells interactions. Acta Pharmaceutica Sinica B.

[72] Saxena M, Yeretssian G (2014). NOD-Like Receptors: Master Regulators of Inflammation and Cancer. Frontiers in Immunology.

[73] Ahn J, Son S, Oliveira SC, Barber GN (2017). STING-Dependent Signaling Underlies IL-10 Controlled Inflammatory Colitis. Cell Reports.

[74] Yang Y, Wang L, Peugnet-González I, Parada-Venegas D, Dijkstra G, Faber KN (2023). cGAS-STING signaling pathway in intestinal homeostasis and diseases. Frontiers in Immunology.

[75] Cheng M, Kanyema MM, Sun Y, Zhao W, Lu Y, Wang J (2023). African Swine Fever Virus L83L Negatively Regulates the cGAS-STING-Mediated IFN-I Pathway by Recruiting Tollip To Promote STING Autophagic Degradation. Journal of Virology.

[76] Li Q, Liu C, Yue R, El-Ashram S, Wang J, He X (2019). cGAS/STING/TBK1/IRF3 Signaling Pathway Activates BMDCs Maturation Following Mycobacterium bovis Infection. International Journal of Molecular Sciences.

[77] Liu N, Pang X, Zhang H, Ji P (2022). The cGAS-STING Pathway in Bacterial Infection and Bacterial Immunity. Frontiers in Immunology.

[78] Shmuel-Galia L, Humphries F, Lei X, Ceglia S, Wilson R, Jiang Z (2021). Dysbiosis exacerbates colitis by promoting ubiquitination and accumulation of the innate immune adaptor STING in myeloid cells. Immunity.

[79] Tang D, Kang R, Xiao W, Wang H, Calderwood SK, Xiao X (2007). The Anti-inflammatory Effects of Heat Shock Protein 72 Involve Inhibition of High-Mobility-Group Box 1 Release and Proinflammatory Function in Macrophages. The Journal of Immunology.

[80] Liu H-Y, Gu F, Zhu C, Yuan L, Zhu C, Zhu M (2022). Epithelial Heat Shock Proteins Mediate the Protective Effects of Limosilactobacillus reuteri in Dextran Sulfate Sodium-Induced Colitis. Frontiers in Immunology.

[81] Wu PE, Juurlink DN (2017). Clinical Review: Loperamide Toxicity. Annals of Emergency Medicine.

[82] Szilagyi A, Shrier I (2001). Systematic review: the use of somatostatin or octreotide in refractory diarrhoea. Aliment Pharmacol Ther.

[83] Bornschein J, Drozdov I, Malfertheiner P (2009). Octreotide LAR: safety and tolerability issues. Expert Opinion on Drug Safety.

[84] Pohlmann PR, Graham D, Wu T, Ottaviano Y, Mohebtash M, Kurian S (2022). HALT-D: a randomized open-label phase II study of crofelemer for the prevention of chemotherapy-induced diarrhea in patients with HER2-positive breast cancer receiving trastuzumab, pertuzumab, and a taxane. Breast Cancer Research and Treatment.

[85] Rosenfeldt V, Benfeldt E, Valerius NH, Pærregaard A, Michaelsen KF (2004). Effect of probiotics on gastrointestinal symptoms and small intestinal permeability in children with atopic dermatitis. The Journal of Pediatrics.

[86] Barroso FAL, De Jesus LCL, De Castro CP, Batista VL, Ferreira Ê, Fernandes RS (2021). Intake of Lactobacillus delbrueckii (pExu:hsp65) Prevents the Inflammation and the Disorganization of the Intestinal Mucosa in a Mouse Model of Mucositis. Microorganisms.

[87] Yeung C-Y, Chan W-T, Jiang C-B, Cheng M-L, Liu C-Y, Chang S-W (2015). Amelioration of Chemotherapy-Induced Intestinal Mucositis by Orally Administered Probiotics in a Mouse Model. PLOS ONE.

[88] Mack DR (2003). Extracellular MUC3 mucin secretion follows adherence of Lactobacillus strains to intestinal epithelial cells *in vitro*. Gut.

[89] Tian Y, Li M, Song W, Jiang R, Li Y (2019). Effects of probiotics on chemotherapy in patients with lung cancer. Oncol Lett.

[90] Shen S-R, Chen W-J, Chu H-F, Wu S-H, Wang Y-R, Shen T-L (2021). Amelioration of 5-fluorouracil-induced intestinal mucositis by Streptococcus thermophilus ST4 in a mouse model. PLOS ONE.

[91] Ting Z, Yu-hong D, Chu-hui L, Xin-xin C, Hai-xia J, Xiao-wu H (2024). A randomized trial of Bacteroides fragilis 839 on preventing chemotherapy-induced myelosuppression and gastrointestinal adverse effects in breast cancer patients. Asia Pacific Journal of Clinical Nutrition.

[92] Reyna-Figueroa J, Barrón-Calvillo E, García-Parra C, Galindo-Delgado P, Contreras-Ochoa C, Lagunas-Martínez A (2019). Probiotic Supplementation Decreases Chemotherapy-induced Gastrointestinal Side Effects in Patients With Acute Leukemia. Journal of Pediatric Hematology/Oncology.

[93] Wei H, Yue Z, Han J, Chen P, Xie K, Sun Y (2024). Oral compound probiotic supplements can improve the quality of life for patients with lung cancer during chemotherapy: A randomized placebo-controlled study. Thoracic Cancer.

[94] Mego M, Chovanec J, Vochyanova-Andrezalova I, Konkolovsky P, Mikulova M, Reckova M (2015). Prevention of irinotecan induced diarrhea by probiotics: A randomized double blind, placebo controlled pilot study. Complementary Therapies in Medicine.

[95] Boyle RJ, Robins-Browne RM, Tang ML (2006). Probiotic use in clinical practice: what are the risks?. The American Journal of Clinical Nutrition.

[96] Iannitti T, Palmieri B (2010). Therapeutical use of probiotic formulations in clinical practice. Clinical Nutrition.

[97] Eghbali A, Ghaffari K, Khalilpour A, Afzal RR, Eghbali A, Ghasemi A (2023). The effects of LactoCare synbiotic administration on chemotherapy-induced nausea, vomiting, diarrhea, and constipation in children with ALL: A double-blind randomized clinical trial. Pediatric Blood & Cancer.

[98] Zaharuddin L, Mokhtar NM, Muhammad Nawawi KN, Raja Ali RA (2019). A randomized double-blind placebo-controlled trial of probiotics in post-surgical colorectal cancer. BMC Gastroenterology.

[99] Khoruts A, Sadowsky MJ (2016). Understanding the mechanisms of faecal microbiota transplantation. Nature Reviews Gastroenterology & Hepatology.

[100] Li N (2014). Gut microbiota disorders and fecal microbiota transplantation. Parenteral & Enteral Nutrition.

[101] Gui G, Yi J, Zhang S, Xie P (2021). Clinical Efficacy of Fecal Microbiota Transplantation Therapy in the Treatment of Refractory Diarrhea Associated with FOLFIRI Chemotherapy Regimen for Advanced Colorectal Cancer. Medical Innovation of China.

[102] Chang C-W, Lee H-C, Li L-H, Chiang Chiau J-S, Wang T-E, Chuang W-H (2020). Fecal Microbiota Transplantation Prevents Intestinal Injury, Upregulation of Toll-Like Receptors, and 5-Fluorouracil/Oxaliplatin-Induced Toxicity in Colorectal Cancer. International Journal of Molecular Sciences.

[103] Wang S, Xu M, Wang W, Cao X, Piao M, Khan S (2016). Systematic Review: Adverse Events of Fecal Microbiota Transplantation. PLOS ONE.

[104] Marcella C, Cui B, Kelly CR, Ianiro G, Cammarota G, Zhang F (2021). Systematic review: the global incidence of faecal microbiota transplantation-related adverse events from 2000 to 2020. Alimentary Pharmacology & Therapeutics.

